# Prediction of 90-day mortality risk after unplanned emergency department visits of advanced stage cancer patients

**DOI:** 10.1007/s00520-024-08919-z

**Published:** 2024-10-16

**Authors:** Georg Jeryczynski, Christoph Krall, Sabina Pasalic, Dominikus Huber, Filippo Cacioppo, Rupert Bartsch, Thorsten Fuereder, Anton Laggner, Matthias Preusser, Christoph Minichsdorfer

**Affiliations:** 1 Division of Oncology, Department of Medicine I, Medical University of Vienna, Vienna, Austria; 2 Centre for Medical Statistics, Informatics, and Intelligent Systems, Institute of Clinical Biometrics, Medical University of Vienna, Vienna, Austria; 3 Department of Emergency Medicine, Medical University of Vienna, Vienna, Austria

**Keywords:** Emergency department visit, 90-day mortality, Albumin, BMI

## Abstract

**Purpose:**

Cancer represents the leading cause of mortality in high-income countries. In the last years, the rate of emergency department (ED) visits by cancer patients has increased 5.5-fold. These ED visits impose a significant economic burden and may indicate the progression of the oncologic disease. The goal of this retrospective study was to identify patient-derived risk factors, especially focusing on serum albumin and body mass index (BMI) for 90-day mortality following unplanned ED visits by cancer patients.

**Methods:**

A retrospective chart review of all patients with an ICD-10 diagnosis for cancer undergoing palliative treatment presenting at the ED between 2016 and 2018 at the General Hospital of Vienna was performed. Laboratory values, emergency severity index (ESI), and BMI were collected at the ED presentation. 90-day mortality (90MM) was calculated from the ED presentation.

**Results:**

A total of 448 cancer patients were included. Lung cancer (19.2%) and pancreaticobiliary cancer (15.6%) were the most frequent diagnoses. The main reasons for ED visits were pain (20.5%) and fever (17.4%). Sixty-nine percent of patients had to be admitted and 17.5% of patients died during hospitalization. 90MM was highest for patients with low albumin (< 35 g/L vs. > 35 g/L: 60.4% vs. 31.4%; *p* < .0001). When incorporating albumin levels and BMI, patients with both values below the cutoff had the highest risk for death (HR 4.01, 95% CI 2.30–7.02).

**Conclusion:**

Cancer patients face a high risk for hospitalization when presenting at the ED. The 90MM rate is highest in patients with low BMI and albumin levels. This highlights an especially vulnerable cohort of cancer patients for whom supportive care and palliative care have to be optimized.

## Introduction

Despite a slight decline in incidence rates, cancer is the leading cause of death in high-income countries [1–3]. Cancer patients undergoing antineoplastic therapy are at increased risk of adverse events leading to unplanned emergency department visits [4]. According to observational studies, 50% of cancer patients receiving antineoplastic therapy will be hospitalized at some point [5].

Breast, lung, gastrointestinal (GI) tract and prostate cancer represent the most common cancer diagnoses at the emergency department (ED) [4, 6–8]. The most frequent reasons for ED visits for cancer patients are respiratory distress, pain, GI symptoms, and fever [6–8]. In the last 10 years, the rate of emergency department visits due to complications of radiation therapy or systemic treatment has increased 5.5-fold [9]. Interestingly, hospitalization rates of cancer patients at the ED are significantly higher for “real world patients” compared to patients in randomized clinical trials (51% vs. 16%) [5]. Studies estimate that around 50% of unplanned ED visits of cancer patients can be preventable and are merely an expression of insufficient supportive care, especially in patients with advanced cancer [10–12].

Over 90% of treatment-related emergency visits by cancer patients ultimately lead to hospital admission and around 5% of these patients die during hospitalization [9].

Besides the high admission- and in-patient death rate, ED visits by oncologic patients represent a significant economic load for health care systems worldwide. In 2007, it was calculated that the cost for a chemotherapy-related ED visit was approximately $ 800. However, an inpatient treatment was estimated to cost roughly $ 22.000 [13]. Besides the financial burden, adverse events leading to unplanned ED visits and inpatient treatments may cause delays in chemotherapy administration, which can have detrimental effects on the survival of cancer patients [14].

Unplanned ED visits by cancer patients either due to therapy or cancer-related complications will not be fully preventable. Given the high frequency of ED visits and their burden on health care systems, it would be beneficial to guide the ED and inpatient facility resources to cancer patients at the highest risk for a detrimental outcome.

It is of paramount importance to identify cancer patients at the ED with a higher risk for an adverse outcome, thus enabling health care providers to adequately allocate resources. Both, low albumin and low BMI are associated with patient’s nutritional status, increased adverse events of systemic cancer therapy, and decreased survival [15–18]. Albumin and BMI are parameters that are easy to assess and obtain in an emergency medicine setting.

The goal of this retrospective pilot study was to identify easily accessible patient-derived risk factors for 90-day mortality after an unplanned ED visit. We aimed to investigate the influence of body mass index (BMI) and serum albumin at ED presentation on the mortality risk of advanced cancer patients at the ED.

## Methods

We performed a retrospective chart review of all patients with a documented oncological diagnosis according to ICD-10 coding that was presented at the Department of Emergency Medicine of the General Hospital Vienna (AKH Wien) between August 2016 and July 2018. Patients who did not receive treatment at the AKH Wien or were treated at a Department other than the Department for Medicine I, Division of Oncology, were excluded. We also excluded patients in a curative setting and such patients who were regularly seen by physicians of our division but did not receive antitumor treatment (i.e., best supportive care, watch and wait). Active antitumor treatment was defined as regular administration of antitumoral substances through the outpatient infusion clinic or the inpatient ward or, in the case of oral antitumor agents, regular checkups at our outpatient department within the last 90 days before presenting at the emergency department. In patients who presented more than once at the emergency department, we focused on the first visit.

Information on tumor entity, type of treatment, as well as reasons for presentation were collected. Presenting symptoms were grouped into several subcategories as was the final diagnosis at the time of discharge. We also assessed whether the presentation was related to the tumor, the antitumor treatment, or for unrelated reasons. In case the date of death was not documented in our hospital documentation system, the insurance data check-up was performed (cutoff date 2022/06/20) to calculate 90-day mortality. Laboratory values, emergency severity index (ESI), and BMI were collected at the time of presentation.

### Statistical methods

Qualitative variables are reported by absolute and relative frequencies, and quantitative variables by median and range. Correlations between 90-day mortality with single covariates were investigated with Chi-squared tests, and a logistic regression model for 90-day mortality with covariates albumin, BMI, sex, and oncological diagnosis was analyzed. Combined effects of low BMI and low albumin were analyzed by first introducing a new variable counting how many of the two values were lowered (0, 1, or 2), and then comparing Kaplan–Meier curves for each pair of values by log-rank tests with Bonferroni-Holm adjustment. A two-sided *p*-value of less than 0.05 was considered significant.

## Results

A total of 2870 patients with cancer were identified. After the application of exclusion criteria, 448 patients were included in the final analysis. Median age was 64.5 (range 18–92) years, and 203 (45.3%) of patients were female. The most common oncological diagnoses were lung cancer (19.2%), pancreatobiliary carcinoma (15.6%), and colorectal cancer (11.2%). Almost half of the patients were treated in the setting of the outpatient infusion clinic (68.1%), either with chemotherapy alone (47.3%) or with the combination of chemotherapy and monoclonal antibodies (13.8%). In total, 42.4% of emergency department visits were due to problems related to the tumor itself, 22.1% to the antitumor therapy, and 35.5% to problems considered unrelated to the underlying neoplasia. The most common presenting complaints were pain (20.5%), fever (17.4%), or respiratory problems (14.7%). A summary can be found in Table 1. Sixty-nine percent of patients had to be admitted, while 31.0% could be discharged to home care. The majority of patients were discharged with a diagnosis of infection (23.4%) or tumor progression (17.6%). Problems related to pain or gastrointestinal symptoms (i.e., diarrhea, nausea/vomiting, and constipation) could be most frequently handled in an outpatient setting (51.1% and 38.8%, respectively).

**Table 1:** Baseline characteristics and distribution of all variables by patient according to their management. *BMI* body mass index, *ESI* emergency severity index

	Discharged from ED	Required admission	Total (% of total)
	139 (31.0%)	309 (69.0%)	448 (100%)
**Sex**			
Male	72 (51.8%)	173 (56.0%)	245 (54.7%)
Female	67 (48.2%)	136 (44.0%)	203 (45.3%)
Age (median, range)	62 (19–86)	66 (20–92)	65 (19–92)
**Oncological diagnosis**			
Lung	21 (24.4%)	65 (75.6%)	86 (19.2%)
Pancreatobiliary	20 (28.6%)	50 (71.4%)	70 (15.6%)
Colorectal	20 (40.0%)	30 (60.0%)	50 (11.2%)
Breast	22 (48.9%)	23 (51.1%)	45 (10.0%)
Head/neck	6 (17.1%)	29 (82.9%)	35 (7.8%)
Sarcoma	10 (33.3%)	20 (66.7%)	30 (6.7%)
Upper GI	2 (8.7%)	21 (91.3%)	23 (5.1%)
Lymphoma/multiple myeloma	9 (40.9%)	13 (59.1%)	22 (4.9%)
Prim. CNS	13 (59.1%)	9 (40.9%)	22 (4.9%)
Renal cell carcinoma	6 (33.3%)	12 (66.7%)	18 (4.0%)
Neuroendocrine	4 (36.4%)	7 (63.6%)	11 (2.5%)
Other	6 (16.7%)	30 (83.3%)	36 (8.0%)
**Presenting symptom**			
Pain	47 (51.1%)	45 (48.9%)	92 (20.5%)
Fever	14 (17.9%)	64 (82.1%)	78 (17.4%)
Respiratory	16 (24.2%)	50 (75.8%)	66 (14.7%)
Deterioration of general condition	6 (11.5%)	46 (88.5%)	52 (11.6%)
Diarrhea/nausea/vomiting/constipation	19 (38.8%)	30 (61.2%)	49 (10.9%)
Neurological symptoms	15 (32.6%)	31 (67.4%)	46 (10.3%)
Bleeding	7 (30.4%)	16 (69.6%)	23 (5.1%)
Cardiological symptom	5 (45.5%)	6 (54.5%)	11 (2.5%)
Edema/effusion	3 (37.5%)	5 (62.5%)	8 (1.8%)
Urological symptoms	2 (33.3%)	4 (66.7%)	6 (1.3%)
Local problem at tumor site	1 (20.0%)	4 (80.0%)	5 (1.1%)
Medical device	2 (66.7%)	1 (33.3%)	3 (0.7%)
Other	2 (22.2%)	7 (77.8%)	9 (2.0%)
**Discharge diagnosis**			
Infection	20 (19.0%)	85 (81.0%)	105 (23.4%)
Tumor progression	21 (26.6%)	58 (73.4%)	79 (17.6%)
Pain	25 (67.6%)	12 (32.4%)	37 (8.3%)
Diarrhea/nausea/vomiting/constipation	15 (44.1%)	19 (55.9%)	34 (7.6%)
Edema/effusion	7 (31.8%)	15 (68.2%)	22 (4.9%)
Cardiological symptom	7 (38.9%)	11 (61.1%)	18 (4.0%)
Bleeding	4 (23.5%)	13 (76.5%)	17 (3.8%)
Blood clot/thrombosis	5 (29.4%)	12 (70.6%)	17 (3.8%)
Surgical	1 (6.3%)	15 (93.8%)	16 (3.6%)
Neurological	8 (57.1%)	6 (42.9%)	14 (3.1%)
Hematological toxicity	2 (15.4%)	11 (84.6%)	13 (2.9%)
Exsiccosis	4 (30.8%)	9 (69.2%)	13 (2.9%)
Respiratory symptom	5 (50.0%)	5 (50.0%)	10 (2.2%)
Acute kidney injury	1 (11.1%)	8 (88.9%)	9 (2.0%)
Medical device problem	2 (22.2%)	7 (77.8%)	9 (2.0%)
Drug related problem	2 (40.0%)	3 (60.0%)	5 (1.1%)
Local problem at tumor site	1 (25.0%)	3 (75.0%)	4 (0.9%)
Urological symptom	1 (25.0%)	3 (75.0%)	4 (0.9%)
Deterioration of general condition	1 (33.3%)	2 (66.7%)	3 (0.7%)
Other	7 (36.8%)	12 (63.2%)	19 (4.2%)
**Association**			
Tumor	54 (28.4%)	136 (71.6%)	190 (42.4%)
Therapy	29 (29.3%)	70 (70.7%)	99 (22.1%)
Other	56 (35.2%)	103 (64.8%)	159 (35.5%)
BMI (median, range)	24.7 (13.3–42.9)	23.0 (14.5–39.1)	23.5 (13.3–42.9)
Albumin g/L (median, range)	39.3 (16.8–47.0)	34.45 (16.8–46.2)	35.7 (16.8–47.0)
**ESI**			
1	0 (0.0%)	2 (100.0%)	2 (0.4%)
2	29 (21.0%)	109 (79.0%)	138 (30.8%)
3	91 (37.9%)	149 (62.1%)	240 (53.6%)
4	8 (47.1%)	9 (52.9%)	17 (3.8%)
Missing	11 (21.6%)	40 (78.4%)	51 (11.4%)
90-day mortality	31 (22.3%)	154 (49.8%)	185 (41.3%)

Median time between the last contact and the time of presentation at the emergency department was 7, 10, and 8 days for patients treated at the outpatient infusion clinic, the inpatient ward, and the outpatient department, respectively. In patients who had to be admitted, the median duration of admission was 8 days (0–89).

Of patients who had to be admitted, 54 (17.5%) patients died while being an inpatient. This proportion was highest in patients admitted for a tumor-related problem (48 of 136 (35.3%)) While the 90-day mortality rate in inpatients was 49.8%, it was only 22.3% in outpatients. Tumor-related cases had the highest 90-day mortality rate among both groups (29.5% in inpatients, 38.8% for outpatients).

ESI and BMI were available in 390 and 398 patients, respectively. Amongst them, the most common ESI grading was 3 (61.5%).

### Impact of albumin and BMI on 90-day mortality

The median BMI was 23.5 (13.3–42.9). Albumin levels at the time of presentation were available in 355 (81.7%) patients; median albumin was at 35.7 g/L (16.8–47.0).

Patients with low (< 35 g/L) albumin levels showed higher 90-day mortality than those with high levels (> 35 g/L; 60.4% vs. 31.4%, *p* < 0.0001). No difference in 90-day mortality between weight classes was identified by the chi-squared test (low BMI 55.9%, normal BMI 42.9%, high BMI 39.5%, *p* = 0.22). However, Kaplan–Meier curves show separation between the three curves, and lower hazards were observed in patients with normal (HR 0.67, 95% CI 0.46–0.96) and high (HR 0.55, 95% CI 0.38–0.81) BMI than patients with low BMI. Log-rank tests with Bonferroni-Holm adjustment only identify a difference between high and low BMI (*p* = 0.0065). Unlike BMI and albumin levels, age below or above 65 years was not a predictor of 90-day mortality (43.7% vs. 38.4%, *p* = 0.2972).

**Fig. 1:**
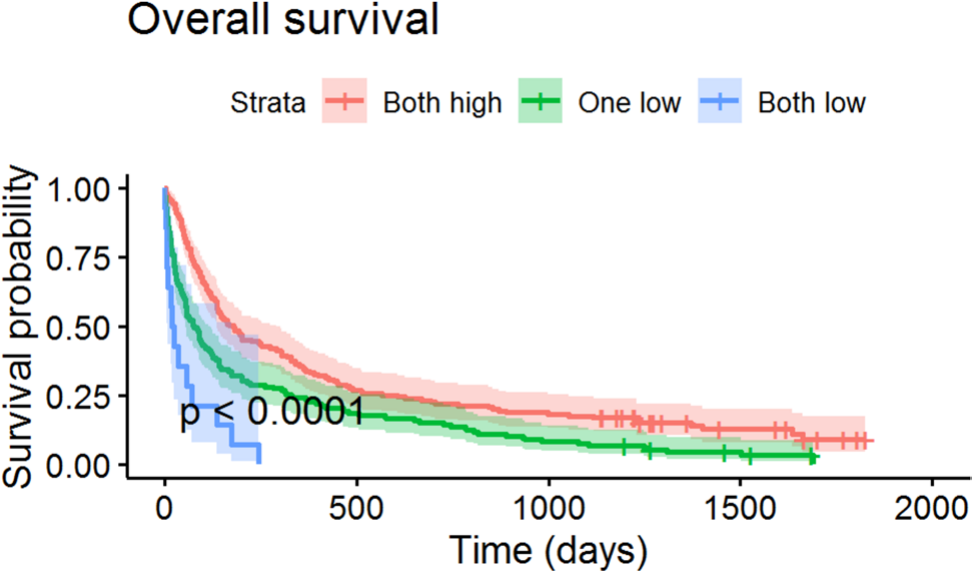
Kaplan Meier curves for patients with neither albumin nor BMI lowered (“both high”), one or both values decreased

We then classified patients according to whether both albumin and BMI were under the critical values, whether one of both values was below the cutoff, or none of them. Pairwise differences identified significant differences in 90-day mortality between patients with both values high and patients with one value low (32.7% vs. 56.6%, *p* < 0.01) and between patients with both values high and patients with both values low (32.7% vs. 78.6%, *p* < 0.01), but not between patients with one or two values low (*p* = 0.189). Regarding overall survival, hazards are higher for one or both levels low than for both levels high (one low HR 1.61, 95% CI 1.27–2.04; both low HR 4.01, 95% CI 2.30–7.02). Log-rank tests with Bonferroni-Holm adjustment identified differences between all three pairs of groups (Fig. 1).

No difference between sexes in BMI (*p* = 0.473) or albumin levels (*p* = 0.072) could be identified by *t*-tests. BMI and albumin are weakly correlated (*r* = 0.16, *p* = 0.004). We performed a logistic regression model for 90-day mortality with free variables sex, BMI (classified into low, normal, and high), albumin (classified as low or high), and tumor diagnosis with lung as the reference group due to the highest complete case number (*n* = 86; estimated 90-day mortality, 0.45; 95% CI 0.35–0.56). High albumin levels and female sex conferred a better 90-day mortality. No significant difference was identified for any tumor diagnosis, with the exception of lymphoma/multiple myeloma, which had lower 90-day mortality (est, 0.18; 95% CI [0.07, 0.39], Table 2).

**Table 2:** Logistic regression of 90-day mortality on albumin, BMI, and sex

	OR	95% CI	*p* value
(Intercept)	2.874	[0.985, 8.756]	0.057
Albumin > 35 g/L	0.312	[0.186, 0.515]	0
BMI normal	0.795	[0.303, 2.041]	0.634
BMI high	0.716	[0.263, 1.913]	0.505
Female sex	0.531	[0.313, 0.892]	0.018
Breast cancer	1.63	[0.607, 4.368]	0.329
Colorectal cancer	0.803	[0.312, 2.023]	0.643
Upper GI cancer	0.959	[0.321, 2.909]	0.94
Head and neck cancer	1.563	[0.6, 4.205]	0.365
Lymphoma/multiple myeloma	0.137	[0.02, 0.569]	0.015
Neuroendocrine carcinomas	0.541	[0.069, 2.955]	0.502
Pancreatobiliary cancer	0.697	[0.327, 1.468]	0.344
Primary CNS tumors	0.195	[0.01, 1.141]	0.133
Renal cell carcinoma	0.552	[0.098, 2.695]	0.469
Sarcoma	2.047	[0.722, 6.103]	0.184
Other	1.089	[0.417, 2.878]	0.862

Earlier analyses have shown that a significant proportion of unplanned ED visits could be preventable [19, 20]. While individual approaches of the studies varied, presenting complaints such as pain and gastrointestinal symptoms like nausea, vomiting, diarrhea, or constipation were generally considered preventable, since their occurrence could be avoided through patient counseling and intensified supportive care. Applying this to our cohort, 141 (31.5%) of ED visits may have been avoidable. When applying our model to only these patients (both parameters were known in 79 patients with preventable complaints), more patients with one or two low parameters had to be admitted to inpatient (Table 3). This difference reached significance between patients with both values high when compared with patients with one value below the cutoff (*p* = 0.034).

**Table 3:** Patients with preventable diagnosis and their ED handling according to levels of albumin and BMI (both parameters were known in 79 patients)

	Discharge from ED	Required admission
Both high	18 (78.3%)	26 (46.4%)
One low	5 (21.7%)	27 (48.2%)
Both low	0 (0.0%)	3 (5.4%)

## Discussion

Unplanned ED visits are a frequent occurrence in cancer patients. This puts a significant strain on ED services and medical services. It also reflects the poor experience many cancer patients have with adverse events during their antitumor treatment.

This analysis from a cohort of cancer patients undergoing palliative antineoplastic treatment at a tertiary care center highlights several points. Firstly, our data corroborate earlier results (4) suggesting that patients in a palliative setting have high admission rates (69%) upon presentation at the ED. Unsurprisingly in this patient cohort, most patients presented due to tumor-related problems, while roughly a fifth of patients presented with complications of antitumor-therapy. Regarding the 35.5% of patients whose presentation was regarded as unrelated (e.g., cardiologic symptoms), a degree of uncertainty remains, whether these problems could still be triggered by the underlying disease. The median time between the last contact and the ED presentation was between 7 and 10 days depending on the setting in which the therapy was admitted. This confirms the general clinical impression that the cancer patients’ vulnerability is highest after the first week after therapy.

Hospital admission correlated with a modest mortality rate of 17.5%; however, 90-day mortality for inpatients was twice as high than in patients discharged to home care (49.8% vs. 22.3%). The highest 90-day mortality was seen in tumor-related cases (most commonly tumor progression).

Given the marked inpatient mortality rate and the high 90-day mortality rate, especially in patients admitted to inpatient care, there is a need for simple yet effective tools to predict outcome in cancer patients requiring acute medical services.

Efforts have been made to apply clinical scores predicting the risk for patients undergoing antineoplastic therapy for unplanned ED visits [11, 21, 22]. As an example, the REDUCE Score incorporates comorbidities (COPD, congestive heart failure, renal failure) and blood parameters (hemoglobin, albumin) and takes prior ED visits (in the past 90 days) into account to highlight cancer patients at increased risk for side effects and ED visits [11]. Other scores that aim to predict the outcome of cancer patients like the (modified) Glasgow prognostic score (m)GPS, that is based on simple, readily available blood parameters CRP and albumin, are only validated to assess survival probability at baseline [23–25].

Further, CRP levels can be volatile and their elevation is multifactorial in the course of the disease, in particular as infection associated with rises in CRP levels is one of the most prevalent reasons for unplanned ED visits of cancer patients [4]. Therefore, we wanted to avoid incorporating CRP levels in our risk model.

Albumin and to a lesser degree low BMI have been recognized as markers of tumor-associated cachexia. In our model, subnormal albumin levels and low BMI were predictive for inferior survival, when compared to high levels and normal or high BMI or patients in which just one of these parameters were subnormal. Whether low albumin per se is a negative prognostic factor as an expression of high tumor burden or liver dysfunction due to metastatic disease, or as a surrogate parameter for cachexia is beyond the scope of this study.

One limitation of this study is its retrospective design, which did not allow us to evaluate our score for the prediction of ED visits by cancer patients.

## Conclusion

In our cohort of 448 cancer patients, low albumin and low BMI were associated with higher 90-day mortality. Taken together, our data identify a cohort of patients who could benefit from more intensive supportive care in order to prevent unplanned ED visits. Further, they may help in selecting patients who would benefit from inpatient admission. In addition, these data may provide initial guidance on whether to discuss best supportive or hospice care in this patient population. However, this goes beyond the role of ED consultations and should be reserved for the treating physicians primarily involved in the oncological therapy.

More studies are urgently needed to predict ED visits and unfavorable outcomes of cancer patients in palliative care.

## Data Availability

Data of this study is available from the corresponding author upon reasonable request.
